# Supplementary material for: A corset function of exoskeletal ECM promotes body elongation in Drosophila
Source: Commun Biol. 2021 Jan 19;4:88. doi: 10.1038/s42003-020-01630-9 (PMC7815793; doi:10.1038/s42003-020-01630-9)
Supplement: Supplementary file 2 — Supplementary Information [file 42003_2020_1630_MOESM2_ESM.pdf]

Supplementary Information

A corset function of exoskeletal ECM promotes body elongation

in *Drosophila*

Reiko Tajiri, Haruhiko Fujiwara and Tetsuya Kojima

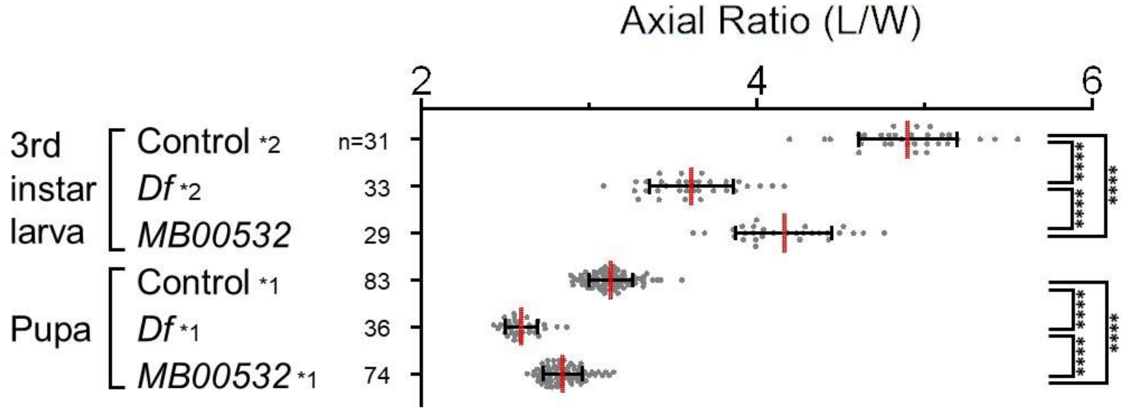

Supplementary Fig. 1. Mean ± S.D. of axial ratios of third instar larval cuticles and pupae of the control and *Cpr11A* mutants. *1, pupal axial ratios shown in Fig. 1k. For control and *MB00532*, male and female axial ratios are compiled. *2, larval cuticle axial ratios shown in Fig. 2f. n, the number of larvae or pupae measured. Significance was assessed using one-way ANOVA ($p<0.0001$) and Tukey's multiple comparisons test. ****$p<0.0001$.

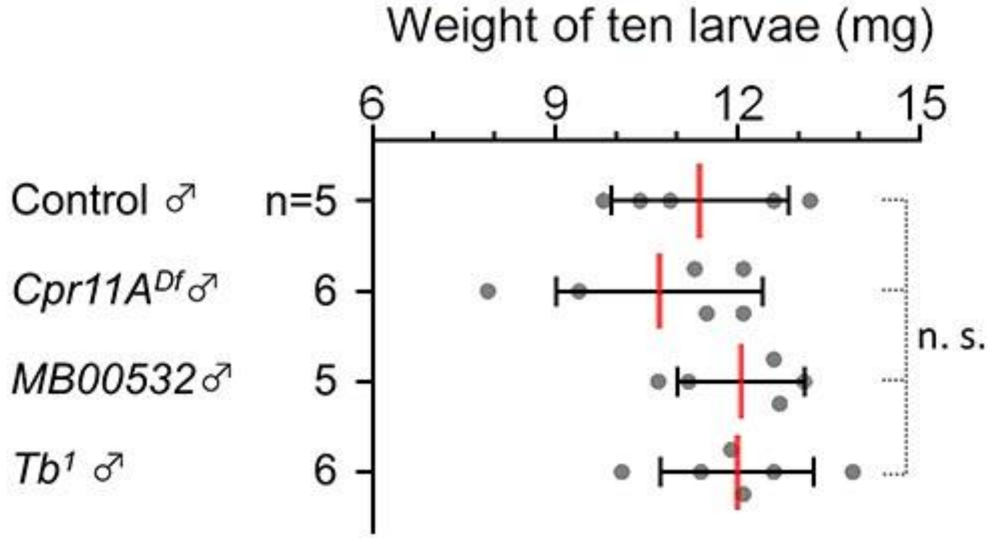

Supplementary Fig. 2. Mean ± S.D. of weights of ten male wandering larvae of control (wild-type), *Cpr11A^{Df}*, *MB00532* and *Tb^{1}*. n, the number of measurements (ten larvae per measurement) for each genotype. No significant difference was found by one-way ANOVA ($p=0.3528$).

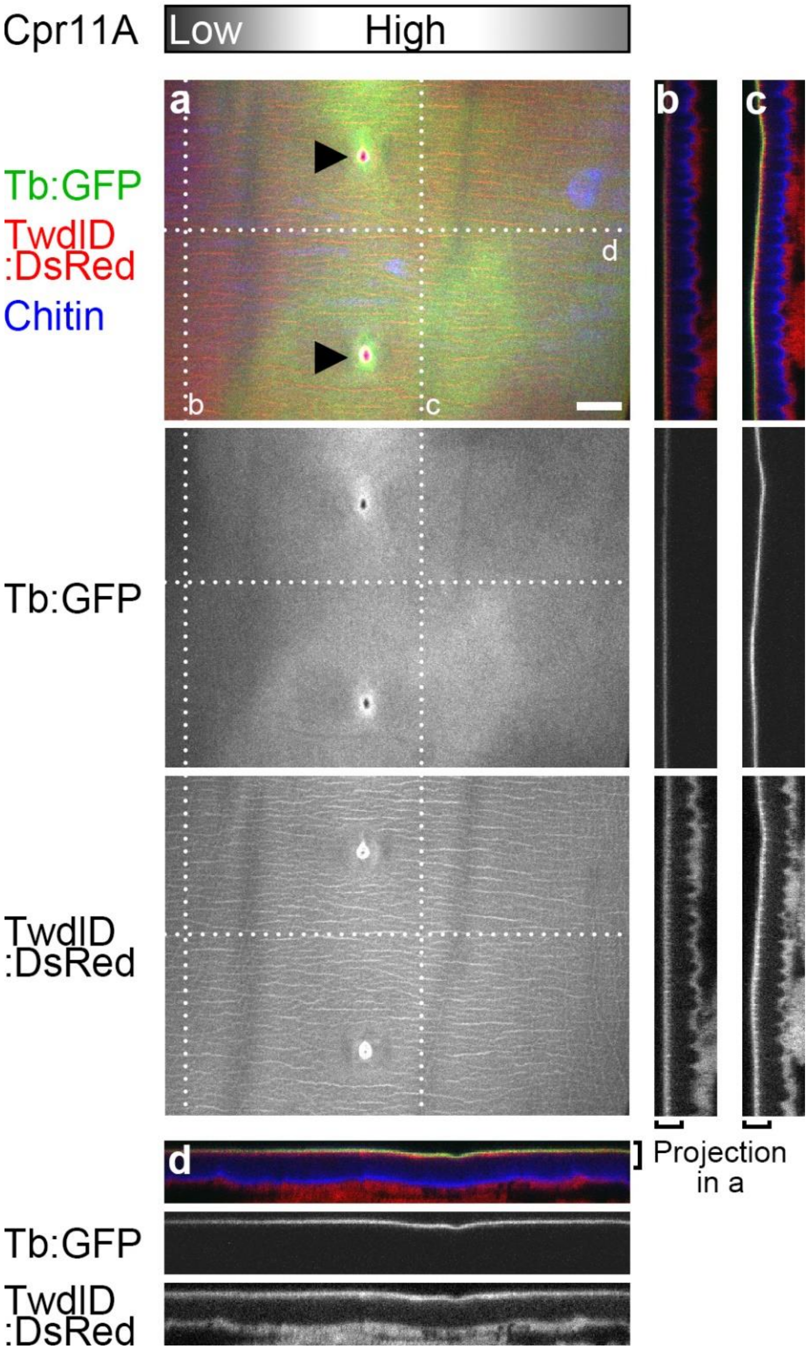

Supplementary Fig. 3. Invariable localization of Twdl proteins in Cpr11A-high and -low areas of a segment. a A projected image of Tb:GFP, TwdlD:DsRed and chitin signals in the cuticle of a dorsal area of abdominal segment 3, equivalent to the area shown in Fig. 4d. **b** A cross-section at the position labeled "b" in (**a**), corresponding to a Cpr11A-low area of the segment. **c** A cross-section at the position labeled "c" in (**a**), corresponding to a Cpr11A-high area. **d** A cross-section at the position labeled "d" in (**a**). Brackets in (**b-d**) indicate the range of focal planes projected in (**a**). Black arrowheads, sensory hairs. Bar, 20µm.

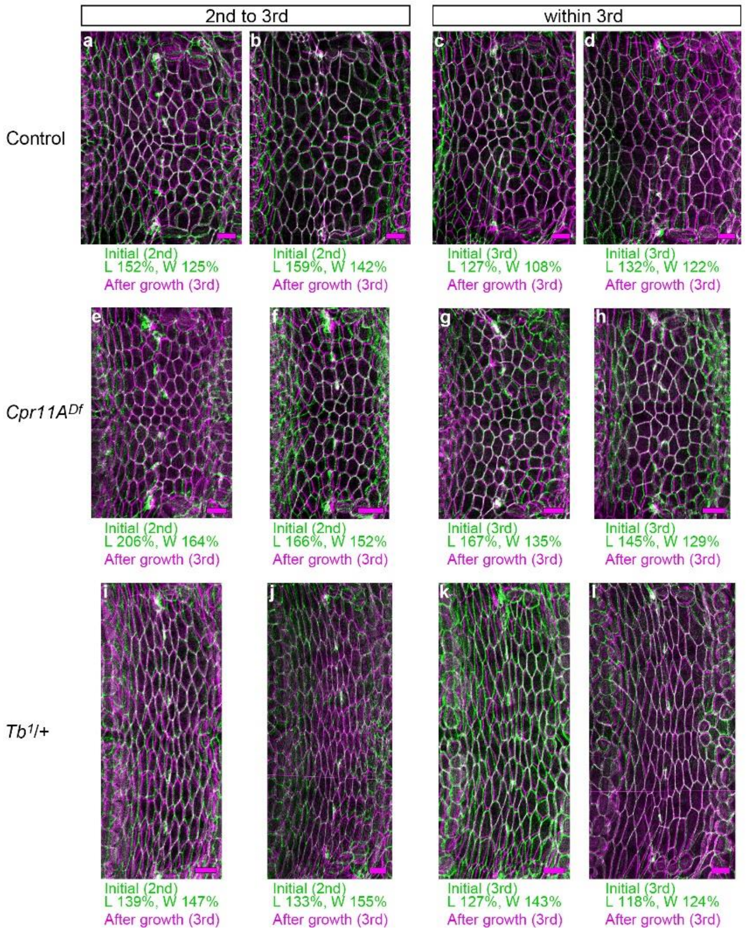

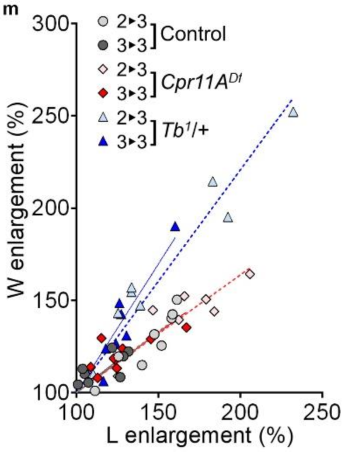

n

Genotype	Stage	n	Linear fit: Label	Slope: Best fit	Slope: CI
Control	2▸3	8	------	0.6617	0.5328 to 0.7907
	3▸3	8	——	0.6980	0.3967 to 0.9994
Cpr11A^Df	2▸3	7	------	0.6383	0.5220 to 0.7545
	3▸3	9	——	0.6510	0.4500 to 0.8520
Tb^1/+	2▸3	8	------	1.208	1.051 to 1.364
	3▸3	7	——	1.394	1.088 to 1.700

Supplementary Fig. 4, related to Fig. 7. Extension of the epidermis during larval growth. a-l More examples of epidermal cell outlines during larval growth. Cell-cell boundaries in the epidermis, marked by Nrg:GFP, on the dorsal side of the abdominal segment 1 (a1) of the control (**a-d**), *Cpr11A^{Df}* (**e-h**) and *Tb^{1}*/+ (**i-l**) larvae. In (**a**, **b**, **e**, **f**, **i**, **j**), changes from the second instar to the third are shown. In (**c**, **d**, **g**, **h**, **k**, **l**), changes within the third instar are shown. The initial images taken at the second instar (**a**, **b**, **e**, **f**, **i**, **j**) or at the third instar (**c**, **d**, **g**, **h**, **k**, **l**) are enlarged along the body length (L) and width (W) by the indicated percentages and shown in green. The post-growth images taken at the third instar are shown in magenta. Bars represent 50μm of the post-growth images. **m** The degrees of enlargements of the initial images that fit the post-growth images. Changes from the second instar to the third (2►3), and changes within the third instar (3►3) of each genotype are plotted. Linear regressions through the point (100%, 100%) are represented by lines. **n** Slopes of the linear regressions. CI, 95% confidence intervals.

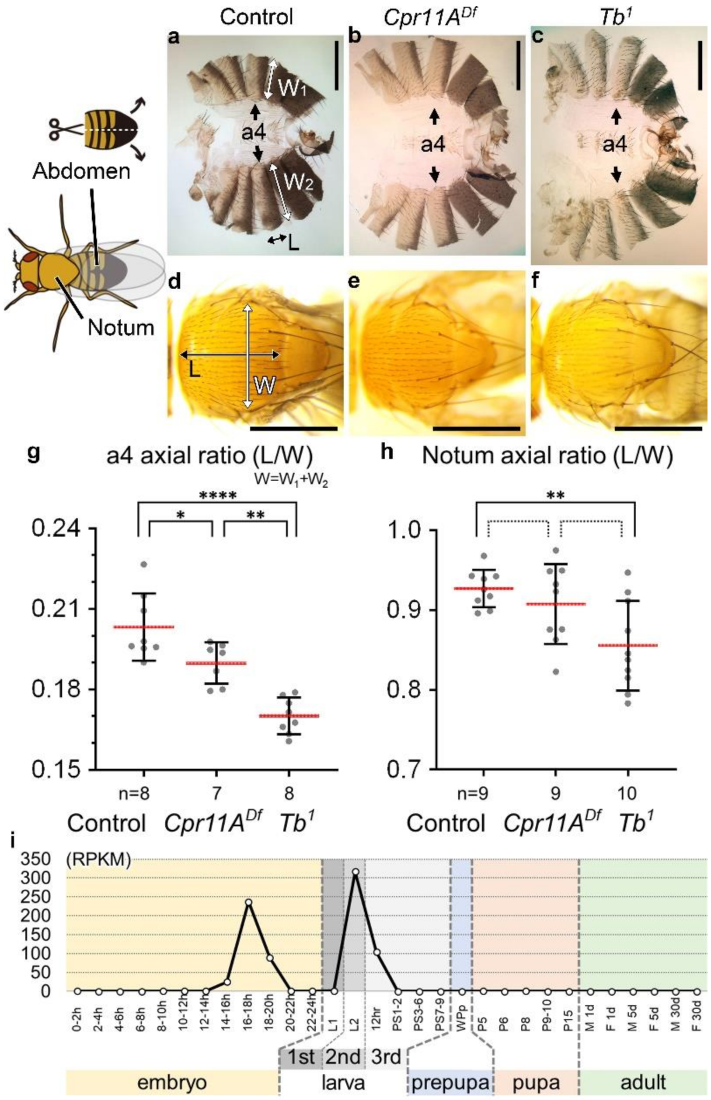

Supplementary Fig. 5. Effects of *Cpr11A*^{Df} and *Tb*^{1} mutations on adult shapes. a-c Adult abdominal cuticles of the control (**a**), *Cpr11A*^{Df} (**b**) and *Tb*^{1} (**c**) males. The length (L) and the width ($W=W_1+W_2$) of the fourth abdominal segment (a4) were measured on each specimen. **d-f** Nota of the control (**d**), *Cpr11A*^{Df} (**e**) and *Tb*^{1} (**f**) males. The notum length (L) and width (W) were measured on each specimen. **g** Mean ± S.D. of a4 axial ratios (L/W) of individual genotypes. n, the number of adults measured for each genotype. Significance was assessed using one-way ANOVA ($p<0.0001$) and Tukey's multiple comparisons test. *p=0.031; **p=0.0017; ****p<0.0001. **h** Mean ± S.D. of notum axial ratios (L/W) of individual genotypes. n, the number of adults measured for each genotype. Significance was assessed using one-way ANOVA (p=0.0065) and Tukey's multiple comparisons test. **p=0.0065; broken brackets, not significant ($p>0.05$). Bars, 0.5mm. **i** The temporal RNA expression profile of *Tb* extracted from modENCODE developmental transcriptome. See Fig. 4a legend for abbreviations.
