# Supplementary material for: A corset function of exoskeletal ECM promotes body elongation in Drosophila
Source: Commun Biol. 2021 Jan 19;4:88. doi: 10.1038/s42003-020-01630-9 (PMC7815793; doi:10.1038/s42003-020-01630-9)
Supplement: Supplementary file 3 — Description of Additional Supplementary Files [file 42003_2020_1630_MOESM3_ESM.pdf]

Description of Additional Supplementary Files

File name: Supplementary Data 1
Description: Source data for Figs. 1k, 2d-f, 3c, e, 5n and Supplementary Figs. 1, 2, 4m and 5g.
